# Diaphragmatic breathing exercises in recovery from fatigue-induced changes in spinal mobility and postural stability: a study protocol

**DOI:** 10.3389/fphys.2023.1220464

**Published:** 2023-06-29

**Authors:** Banafsheh Amiri, Erika Zemková

**Affiliations:** Department of Biological and Medical Sciences, Faculty of Physical Education and Sport, Comenius University in Bratislava, Bratislava, Slovakia

**Keywords:** electromyography, fatigue, low back pain, sedentary adults, trunk and hamstring muscles

## Abstract

Prolonged periods of sitting at work can increase trunk muscle fatigue from the continuous contraction of deep trunk muscles. Insufficient activity of these muscles can decrease muscular support to the spine and increases stress on its passive structures. This can lead to reduced spinal mobility and impaired postural stability. It may also stimulate nociceptor activity leading to pain. However, frequently used recovery modalities such as muscle strengthening and stretching exercises, can be time-consuming, impractical, and difficult to implement in the workplace. Diaphragmatic breathing exercises, which increase the activity of the deep trunk muscles by raising intra-abdominal pressure, seem to be a suitable alternative. However, little is known as to what extent diaphragmatic breathing exercises contribute to the reduction of fatigue induced by prolonged sitting. This paper presents a study protocol that aims to investigate the acute effect of diaphragmatic breathing exercises on recovery of fatigue-induced changes in spinal mobility and postural stability in sedentary middle-aged adults at risk of developing non-specific low back pain. Twenty sedentary adults aged between 25 and 44 years will perform Abt’s fatigue protocol, followed by 1) active recovery using diaphragmatic breathing exercises and 2) passive recovery in the form of lying on the bed, respectively. There will be 1 week of rest in-between. Pre-fatigue, post-fatigue, and after the active and passive recovery, spinal mobility and postural stability will be evaluated using the spinal mouse device and a posturography system, respectively. The electromyography will be used to determine the muscle-fatigue conditions. We hypothesize that active recovery in a form of diaphragmatic breathing exercises would be more effective in restoring spinal mobility and postural stability followed by the fatigue of back and hamstring muscles compared to passive recovery in sedentary adults. Increasing core and respiratory muscle strength via these exercises could be beneficial for overall mobility and stability of the spine. Reducing compressive stress on the passive structures of the spine may be also beneficial for lowering low back pain. Therefore, we believe that diaphragmatic breathing exercises have the possibility to be incorporated into the workplace and contribute to better back health in sedentary middle-aged adults.

**Clinical Trial Registration:** [https://www.irct.ir/trial/67015], identifier [IRCT20221126056606N1].

## 1 Introduction

Modern life have resulted in sedentary behaviors among human societies (Egger et al., 2001; Jans et al., 2007). These behaviors are characterized by low levels of physical activity and an energy expenditure of at least 1.5 metabolic equivalents (Freire et al., 2022). The daily job is a major factor in adults’ sedentary behavior (Kett and Sichting, 2020). Office workers sit for 6.6–10.0 h per day on average (Kett and Sichting, 2020). Continuous contraction of the trunk muscles during prolonged sitting can lead to deep trunk muscle fatigue (Saiklang et al., 2022). Fatigability depends on both the contractile capability of the muscles involved in the task as well as the capacity of the nervous system to provide adequate activation signals to accomplish it (Zemková et al., 2021a). The decreased muscle contractile efficiency and limited capacity of the nervous system can increase articular structural stiffness, and delay muscle response time (Hamaoui et al., 2004). Thus, spinal loading decreases which plays an important role in the mobility of the spine (Yang et al., 2005).

Spinal mobility depends on two anatomical and physiological factors (Hamaoui et al., 2004). The anatomical factor corresponds to articular structural stiffness and determines the range of motion capacity (Hamaoui et al., 2004; Hamaoui et al., 2007). The physiological factor corresponds to muscular excitation (by delayed muscle response time) and determines dynamic mobility capacity (Hamaoui et al., 2004; Hamaoui et al., 2007). These features of the spine may be influenced by prolonged sitting (Hamaoui et al., 2004). While the first one is related to a decrease in the spine’s range of motion, the second one is related to a decrease in spine velocity (Hamaoui et al., 2004). Assessment using the spinal mouse suggests that spinal mobility may be impaired as a result of fatigue induced by prolonged sitting (Lenková and Vasilišinová, 2019). Decreased spinal mobility leads to abnormal lumbar vertebrae movement as a compensatory mechanism (Sung et al., 2014). This exacerbates facet joint instability and increases the risk of experiencing lower back pain (Sung et al., 2014).

Additionally, the combination of decreased muscle contractile efficiency (Tajali et al., 2022), and decreased capacity of the nervous system including insufficient integration of sensory information, and impaired neuromuscular functions under fatigue can alter feedforward and feedback control of postural sway (Akulwar and Mulgaonkar, 2017; Zemková et al., 2021a). For example, fatigued healthy subjects exposed to external perturbations have shown longer activation latencies, an increase in electromyographic amplitude, reduced muscle activity, and increased co-contraction (Zemková et al., 2021a; Zemková et al., 2021b). Also postural control strategies can be altered by acute back muscle fatigue (Johanson et al., 2011). These strategies are similar in healthy individuals when postural demands increased to those used by people with recurrent low back pain. The Zemkova (2021) study discovered that lumbar muscle fatigue can cause alterations in the lumbar spinal curvature, which may play a functional role in explaining the reduced ability to maintain balance when subjected to external perturbations. To measure spinal mobility, the study employed the spinal mouse, while the balance was evaluated using the posturography system (Zemková et al., 2021a). Such an altered postural stability can cause predisposing agents for musculoskeletal disorders, especially low back pain (Hanna et al., 2019; Zhang et al., 2020; Suresh et al., 2021). Due to the fact that low back pain strongly correlates with trunk muscle corset condition (Jabłońska et al., 2021), there is a need to control and recover trunk muscle fatigue.

The muscle fatigue recovery process refers to a return of the functional capacity of body tissues after the onset of fatigue (Yi et al., 2022). Physical exercises play a pivotal role in early intervention and are usually recommended at workplace (Kaeding et al., 2017). Two frequently employed recovery modality types are compensatory exercises and relaxation exercises, both of which can be beneficial for releasing tension and reducing fatigue in the musculoskeletal system (Soares et al., 2019). Compensatory exercises are referred to as short active breaks, involving the discontinuation of job tasks for exercising, and typically include muscle strengthening, flexibility, stretching, and breathing exercises (Soares et al., 2019). Conversely, relaxation exercises are typically performed at the end of the working day and include flexibility, stretching, breathing, and self-massage exercises (Soares et al., 2019). It might also be combined to complementary therapies representing mind-body interventions, such as acupuncture, yoga, Pilates, progressive muscle relaxation and meditation (Soares et al., 2019). Complementary therapies modality seeks to rehabilitate employees with work-related musculoskeletal disorders according to their individual complaints (Soares et al., 2019). Therefore, these exercises do not serve as a primary preventive measure (Soares et al., 2019). Furthermore, it is important to note that the success of a movement-related intervention is reliant on the motivation and adherence of participants (Kaeding et al., 2017). Therefore, short and sharp interventions are recommended as they have been found to achieve high levels of adherence, with an expected compliance rate of approximately 76% (Bell and Burnett, 2009). However, muscle strengthening, stretching, and flexibility exercises can be time-consuming, impractical, and difficult to implement in the workplace. Employers must provide appropriate locations for exercise at adequate intervals to address these issues. To avoid these shortcomings, one can use diaphragmatic breathing exercises during prolonged sitting periods at the workplace. Diaphragmatic breathing involves the contraction of the diaphragm muscle located between the chest and abdomen, which can improve breathing efficiency and oxygen delivery (Dhalla, 2022). Normal respiration, also known as tidal breathing, is driven by a group of muscles known as the “respiratory pump,” with the diaphragm being the major respiratory muscle (Russo et al., 2017). When the diaphragm contracts during normal inspiration, it pushes on the abdomen and causes the lower ribs to expand outwards, generating a trans-diaphragmatic pressure that allows for ventilation of the lungs and gas exchange (Russo et al., 2017). Expiration is generally passive, but the expiratory muscles become active during increased breathing effort. Studies Vostatek et al. (2013); Russo et al. (2017) have shown that optimal respiration requires active control of the diaphragm, with the lower ribs staying low and only expanding laterally during inspiration, while the abdomen expands instead of the chest. Diaphragmatic breathing has been found to facilitate slow respiration, with trained individuals achieving slower respiratory rates and greater diaphragm excursion during slow breathing (Russo et al., 2017). Correct and balanced diaphragm performance has been shown to help maintain abdominal pressure and smooth respiration (Russo et al., 2017; Hamasaki, 2020). Increasing intra-abdominal pressure in turn causes activation of the abdominal wall and pelvic floor muscles (Rasheed et al., 2021). This muscle activation provide posterior and anterolateral and inferior stability, thus increasing overall spinal stability (Rasheed et al., 2021). Kang et al. (2016) reported that intra-abdominal pressure that is produced by the connection of trunk stabilization and respiratory muscles affects waist stability and protects the trunk. Training of respiratory muscles increases muscle firing, proprioception of the diaphragm, low back musculature, deep core musculature, and respiratory muscle strength (Stephens et al., 2017). This can have a positive effect on spinal mobility and postural stability. For example, physiotherapeutic breathing exercises, like yoga and Pilates, have been found equally effective in improving spinal mobility in healthy young women (Csepregi et al., 2022). In addition, Stephens et al. (Stephens et al., 2017) demonstrated improvement of postural stability after 8-week diaphragmatic breathing exercises in healthy persons. The improvement of static and dynamic balance have been also reported in athletes with chronic low back pain (Otadi et al., 2021).

Breathing exercises are also effective in the treatment of musculoskeletal complaints, such as low back pain (Kang et al., 2016; Ahmadnezhad et al., 2020; Otadi et al., 2021). For instance, the activation patterns of trunk muscles change immediately during the lifting task after performing an abdominal drawing-in exercise in subjects with recurrent low back pain (Suehiro et al., 2021). A session of respiratory muscle training can also reduce the activity of some ankle joint muscles while performing overhead squats in athletes with chronic low back pain (Borujeni and Yalfani, 2020).

Although the effect of diaphragmatic, exhalation, and inspiratory breathing exercises on spinal mobility (Kang et al., 2018; Lenková and Vasilišinová, 2019) and postural stability (Tajali et al., 2022; Stephens et al., 2017; Armstrong et al., 2018; D’souza et al., 2021; Farzami and Anbarian, 2020; Roth et al., 2021; Ferraro et al., 2020) has been investigated, there are no studies dealing with their recovery following by prolonged sitting at the workplace. People exposed to repetitive, prolonged sitting at their work are prone of back problems, especially in late middle-age. There is also growing concern over the effects of sedentary lifestyles on the young people’s health. However, prevention programs for them are rare. This paper presents a study protocol that aims to investigate the acute effect of diaphragmatic breathing exercises on recovery of fatigue-induced changes in spinal mobility and postural stability in sedentary middle-aged adults at risk of developing non-specific low back pain.

## 2 Materials and methods

### 2.1 Study design

**FIGURE 1:**
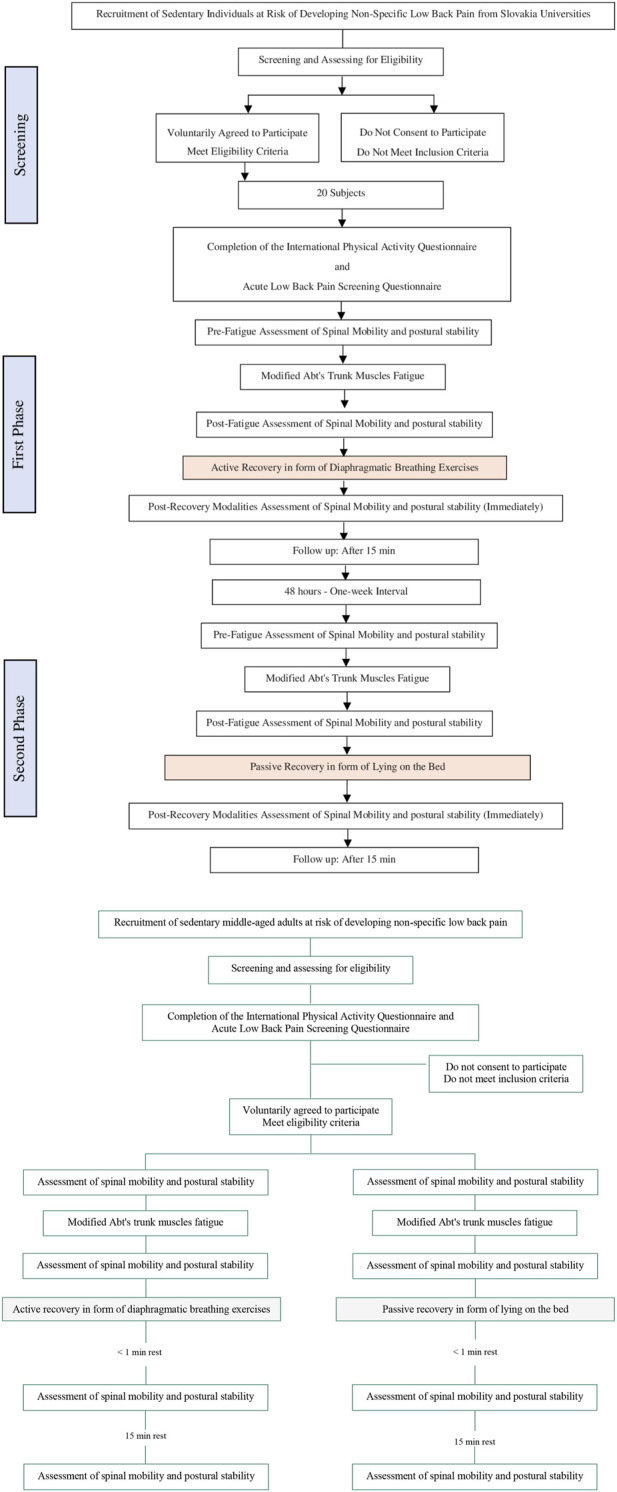
Flow-diagram of the study protocol.

This experimental pre-post study is designed to investigate the acute effect of diaphragmatic breathing exercises on recovery of fatigue-induced changes in spinal mobility and postural stability in sedentary middle-aged adults at risk of developing non-specific low back pain. This is a study protocol that will be carried out and reported in accordance with the Standard Protocol Items: Recommendations for Interventional Trials statement (SPIRIT). Figure 1 illustrates the study design. The study has been approved by the ethics committee of the Faculty of Physical Education and Sport, Comenius University in Bratislava (No. 5/2022) and the Ethics Committee of Kerman University of Medical Sciences (IR.KMU.REC.1401.386). It also has been approved by Iranian Registry of Clinical Trials (registration reference: IRCT20221126056606N1).

### 2.2 Participants and setting

This study will be performed on 20 sedentary individuals aged 25–44 years old, at risk of developing non-specific low back pain, who will mainly be employees of university. This effect size is sufficient to determine the significant effects of diaphragmatic breathing exercises on the recovery of spinal mobility and postural stability due to trunk muscles fatigue.

The present study will be performed at the Faculty of Physical Education and Sport, Comenius University in Bratislava, Slovakia.

#### 2.2.1 Inclusion and exclusion criteria

Inclusion criteria for the participants were established as follows: The sedentary middle-aged adults (aged 25–44 years old) at a risk of developing non-specific low back pain, and self-reporting of sitting for at least 2 hours on any working day (Saiklang et al., 2022). Potential participants will be screened for the study using the international physical activity questionnaire (IPAQ) to recognize those who are sedentary and using the Acute LBP Screening Questionnaire (ALBPSQ) to identify those who are at risk of developing non-specific low back pain. The volunteers will be excluded if they meet any of the following criteria: pregnancy (Abboud et al., 2014); history of spinal, femoral, or intra-abdominal surgery in the previous 12 months (Sitthipornvorakul et al., 2015); have been diagnosed with a congenital anomaly of the spine, arthritis, rheumatoid, infection of the spine and discs, spondylolisthesis, ankylosing spondylitis, spondylosis, tumor, systemic lupus erythematosus, or osteoporosis (Sitthipornvorakul et al., 2015); receiving physiotherapy services 1 month before the beginning of the study (Fortun-Rabadan et al., 2021); having suffered any kind of mental health disorder, including anxiety and depression, during the past 3 years (Fortun-Rabadan et al., 2021).

#### 2.2.2 Sample size estimation and sampling method

A prior sample size estimation was done using the G*Power software package (version 3.1.9.7) based on the study by Fonta et al. (2021). The input parameters were as follows: statistical test = repeated measures, within - between interaction; effect size F = 0.25; α err prob = 0.05; and power (1 − β err prob) = 0.80. As a result, the total sample size was calculated to be 11 subjects. In consideration of the possibility of dropouts, a slightly larger number of subjects will be recruited to participate in this study. The total sample size would be approximately 20 subjects.

A purposeful sampling strategy will be used and participants based on their age, sedentary behavior, and other relevant factors that are related to the study’s focus and have been explained in inclusion and exclusion criteria section will be selected.

### 2.3 Procedures

Each participant will take part in two phases of the experiment including familiarization and data collection.

#### 2.3.1 Familiarization

In the first phase, all subjects will undergo a familiarization session, about 1–2 h before data collection. The study objectives and procedures will be explained to participants and informed consent will be taken from each participant before the study. In the second phase, participants will complete a questionnaire that includes personal and baseline characteristics. Body mass will be measured using a calibrated digital scale, and height will be measured using a single stadiometer. The weight plates used in the trunk muscles fatigue protocol for each participant will be chosen during the familiarization phase. The heaviest weight that participants can perform each exercise 20 times in 40 s in the correct form will be used (Askari and Esmaeili, 2021). During this phase, subjects will be asked to eat their normal meal while avoiding strenuous exercise and performance-enhancing energy drinks for the 48 h prior to data collection sessions (Armstrong et al., 2018).

##### 2.3.1.1 Health and safety considerations

Participants will complete a “health and medical questionnaire” before the study and will be closely monitored by professionals to minimize injury risk. Participants will stop exercises if experience pain or discomfort. Adverse events will be documented and addressed, with modifications to the protocol if necessary.

#### 2.3.2 Data collection

Data from all participants will be collected under three conditions:

1. Pre-fatigue assessment (at baseline),
2. Post-fatigue assessment (immediately after fatigue protocol),
3. Post-recovery modalities assessment (immediately, and 15-min after cessation of active and passive modality, respectively).

Assessing the effects of the intervention at the 15-min follow-up can provide insight into the potential duration of these effects.

All participants will be tested at the same time of day by the same examiners.

The study will involve gathering individual demographic information and outcome data. The study team will enter the questionnaire data into a database and verify its accuracy before using it. All information and outcome data will be kept on password-protected computers that only the authorized members of the study team will be able to access. Data management will outline the procedures for gathering, recording, storing, and archiving data.

##### 2.3.2.1 Primary outcomes for screening

###### 2.3.2.1.1 International physical activity questionnaire

Potential participants will be screened for the study using the International Physical Activity Questionnaire [IPAQ] to recognize those who are sedentary. The IPAQ is a formal self-report tool used to measure physical activity in one’s normal daily routine (Mehta et al., 2018). This questionnaire is available in two versions, long and short (Demircioğlu et al., 2021). The IPAQ short form (IPAQ-SF) is used to screen physical activity levels in the population at large, while the IPAQ long form (IPAQ-LF) is used in research studies or clinical settings to assess physical activity levels (Mehta et al., 2018). In both versions of the questionnaire, the common objective is to assess the amount and intensity of physical activity an individual participates in per week (Mehta et al., 2018). Each version considers four domains: leisure-time physical activity work-related activities, domestic and gardening activities, and transport-related activities. The long interview administered version of the IPAQ, which contains 27 items in the four domains, will be used in the present study (Mehta et al., 2018). Using the long form, it will be possible to calculate domain-specific scores, activity-specific scores, and continuous scores. The continuous scores, expressed in metabolic equivalent minutes (MET), will be a measure of physical activity. According to these scores subjects will be categorized as engaging in low-, moderate-, or high-level physical activity (Mehta et al., 2018).

###### 2.3.2.1.2 Acute low back pain screening questionnaire

The Acute Low Back Pain Screening Questionnaire (ALBPSQ) identifies those who are at risk of developing non-specific low back pain. The questionnaire has been endorsed by guidelines from the New Zealand work health authority (Hanna et al., 2019). The level of back pain will be calculated based on the duration and intensity of the pain. A Likert rating scale from 0 (no pain) to 5 (pain as bad as it can get) will be used to assess perceived beliefs about the psychological impact of pain on the participant. Ratings on the scored items will be subsequently summed to determine a total score and domain sum score. A higher rating indicates a higher level of risk.

##### 2.3.2.2 Secondary outcomes for intervention evaluation

###### 2.3.2.2.1 Assessment of the trunk muscle fatigue

The Delsys Trigno™ wireless EMG system, will be used to record electromyography signals from the trunk and hamstring muscles. These muscles will be the lumbar multifidus at the L5 level, and erector spinae muscles at the L1 level and hamstring muscles. Prior to electrode placement, the skin will be prepared by abrasion and cleaning with alcohol.

For the Multifidus Muscles

⁃ Electrode placement: on the line connecting the caudal tip of the posterior superior iliac spine (SIPS) to the space between L1 and L2, at the level of the spinous process of L5, 2–3 cm from the medial line,
⁃ Inter-electrode distance: 25 mm,
⁃ Clinical test: maximum isometric strength of the back extensor muscles.

For the Longissimus Muscles: erector spinae

⁃ Electrode placement: two fingers apart in a lateral direction from the spinous process L1,
⁃ Inter-electrode distance: 25 mm,
⁃ Clinical test: maximum isometric strength of the back extensor muscles.

For the Hamstring Muscles: medial hamstring

⁃ Electrode placement: The medial hamstring on both lower extremities: the electrode for the medial hamstring will be placed midway between the ischial tuberosity and the medial epicondyle of the tibia,
⁃ Clinical test: maximum isometric strength of the hamstring muscles.

The raw sEMG signals will be recorded at a sampling frequency of 2,000 Hz. Butterworth band-pass filters with a passband of 20 Hz and a stopband of 450 Hz will be applied to remove signals that will not be due to muscle activity. The features of muscle fatigue by EMG are amplitude increase and transformation from a high-frequency spectrum to a low-frequency spectrum. It is assumed that fatigue and recovery are mutually exclusive. Therefore, decrease amplitude and transformation from a low-frequency spectrum to a high-frequency spectrum are features of muscle recovery by EMG. In the present study, the mean amplitude (root mean square, RMS) and the frequency features (mean power frequency, MPF, and median frequency MDF) of the sEMG signals will be used to evaluate muscle fatigue and recovery. To accomplish this, the raw EMG signal will be processed with a fast Fourier transformation to determine the mentioned variables. The fast Fourier transform is a mathematical technique used to transform a time-domain signal into its frequency-domain representation (Mateo and Talavera, 2018).

###### 2.3.2.2.2 Assessment of the spinal mobility

Intersegmental mobility, overall and regional spinal range of motion will be measured in the sagittal plane in the standing position using a Spinal Mouse device (Csuhai et al., 2020). It is a wireless electronic, and non-invasive skin-surface tool for computer-assisted imaging and radiation-free examination (Post and Leferink, 2004). The Spinal Mouse’s reliability and validity have been established in populations that were healthy and symptomatic, including back pain (Topalidou et al., 2014). Before starting the examination, every subject will be registered in Spinal Mouse software with gender, age and randomly allocated study codes. Subjects will be asked to take three different standing positions: relaxed but erect (not corrected), maximal flexion and maximal extension of the spine. After undressing the upper body, spinous processes will be palpated, and C7 and S3 will be marked with a body-marker. Spinal Mouse will run paravertebrally along the spinous processes of marked segments. It makes the system capable of recording the contour of skin above the vertebral bodies in the sagittal plane. Positions include:

1. Neutral in standing: Subject will be asked to maintain a relaxed position with the feet shoulder width apart, with straight knees and arms by the side, looking and facing straight horizontally towards the wall.
2. Maximal flexion in standing: Subject will be asked to flex the trunk with straight knees as far as possible with slow motion from segment to segment, aiming to touch the ground with fingertips.
3. Maximal extension in standing: Subject will be asked to cross arms in front of the chest and extend the trunk as far as possible, keeping the knees straight, without extension of the cervical spine.

No warm-up will be performed before the examination and each test will be done once (Post and Leferink, 2004). The mobility values of the segments, including range of flexion from upright (U-F), range of extension from upright (U-E) and total range from extension to flexion (E-F), will be measured (Csuhai et al., 2020).

###### 2.3.3.2.3 Assessment of the postural stability

Participants will be asked to stand barefoot on a force plate with their arms relaxed comfortably at their sides. They will be instructed to stand in an upright position with their feet abducted at 10° and their heels separated mediolaterally by a distance of 6 cm. A series of trials will be conducted in a randomized order under varied conditions: tandem stance on a force plate with eyes open, tandem stance on a force plate with eyes closed, tandem stance on a foam mat (Airex Balance Pad) placed on the force plate with eyes open, tandem stance on a foam mat (Airex Balance Pad) placed on the force plate with eyes closed (Zemková et al., 2016). Subjects will perform one set of 30s under each condition. Short rest periods break will be allowed after every two trials (Zemková et al., 2021c).

Postural stability will be assessed using a FiTRO Sway Check (FiTRONiC, Bratislava, Slovakia). The system measures the actual force in the corners of the force plate and calculates an instant position of the CoP (sampling rate: 100Hz, 12-bit AD signal conversion, resolution of the CoP position: less than 0.1mm, measuring range: 0–1,000/s, non-linearity: ±0.02%FS, combined error: 0.03%, sensitivity: 2 mV/V ± 0.25%, overload capacity: 150%/sensor). FiTRO Sway Check has been shown to have good to excellent reliability of CoP variables, according to a recent study by Zemkova et al. (2021) (Zemková et al., 2021c). The Romberg quotient (eyes closed/eyes open (EC/EO) sway ratio) will also be calculated. Under unstable conditions, variables of postural stability will be registered by using the FiTRO Sway Check (FiTRONiC, Bratislava, Slovakia). The device consists of a square platform supported by four springs with an elasticity coefficient of 40N/mm. Shifting the CoP in the horizontal plane leads to changes of body weight distribution to the four corners of the platform. Force acting in each corner is calculated as a product of the coefficient of elasticity of the spring used and vertical distance measured by means of a fine sensor. The analogue signals are AD-converted and sampled by computer at the rate of 100Hz. Calculations of instant CoP position is based on force distribution to the four corners of the platform. Basic parameters of postural stability (i.e., mean CoP velocity and mean CoP displacements in medio-lateral and anterior-posterior directions) will be analyzed (Zemková et al., 2021c).

#### 2.3.3 Fatigue protocol

The modified Abt protocol will be used in order to induce fatigue of trunk muscles (Abt et al., 2007). The protocol lasts 32 min and consists of four consecutive cycles of eight exercises. Each set consists of exercises in the following order:1) trunk rotation with a medicine ball in a sitting position, 2) prone static torso extension with a medicine ball, 3) lower torso rotation with a medicine ball in a supine position, 4) sit-ups on the incline bench with a weight plate, 5) lateral side binding with a weight plate, 6) lumbar extension rotation with weighted plate, 7) trunk rotation with weighted pulley resistance in standing position, and 8) supine isometric bridge hold. The selection of weight plates for each subject will be performed on a separate day before testing. The heaviest weight with which subjects can perform each exercise 20 times in 40 s in the correct form will be used. Prior to trunk muscle fatigue protocol, a 10- minutes warm-up will be performed including 5 min of *insitu* warming, and 5 min of aerobic stretching, with an emphasis on the hamstring and trunk muscles. Then fatigue protocol will be started. Subjects will perform 20 repetitions of each exercise in 40 s (each repetition in 2 seconds). A pause of 20 s will be between each exercise. The fatigue protocol will be terminated in two ways: 1) when subjects will be no longer able to continue the fourth set of exercises (the last set) with the correct form, and 2) when the subjects will be unable to perform each repetition in 2 seconds in the last set exercises. To ensure occurrence of fatigue, subjects will be rated on their perceived exertion at the end of each phase of the protocol using the 15-point Borg scale (rank 6–20) (Borg, 1970). The point of six represents the absence of fatigue and point of 17–20 indicates failure to perform exercises. If the subjects in the end of fourth round report number 17 or higher, it means the end of the fatigue protocol. If they will report fewer points, they should perform another round until they report the point 17 (Askari and Esmaeili, 2021).

#### 2.3.4 Recovery modalities

##### 2.3.4.1 Diaphragmatic breathing exercises

Subjects will be in crook lying position. They will be advised to deeply inhale through the nose so that they could see their abdomen expanding. They should hold this position for 5 s and then exhale through mouth. To ensure that the subjects are performing the exercise correctly, they will be instructed to place one hand on their chest and the other on their abdomen. They will be advised to breathe in so deeply that they should feel only the movement of the hand on their abdomen and not that of the hand on their chest. Each subject will perform the assigned exercise 10 times per session. They will take 1-min rest between each session (Rasheed et al., 2021).

##### 2.3.4.2 Passive recovery

The subjects will be asked to lie on a bed in a darkened room and do nothing for 24 min after Abt’s trunk muscle fatigue protocol (Seidi et al., 2019).

### 2.4 Statistical analysis

Statistical analyses will be carried out using SPSS Statistics (SPSS Statistics Version 24; IBM Corporation^©^, United States). Prior to statistical comparisons, a Shapiro-Wilk test of normality will be performed for all variables. Not normally distributed data will be analyzed using nonparametric tests. Friedman’s test followed by Dunn’s *post hoc* test will be used for the comparison of the spinal mobility, postural stability and EMG variables at different assessment times (at baseline, immediately after Abt’s fatigue protocol, immediately after recovery modalities, 15-min after recovery modalities, 30-min after recovery modalities). The Kruskal–Walli’s test will be used for the inter-group comparisons (experimental group A vs. experimental group B) at each assessment. For the normally distributed data, a repeated measures Analysis of Variance (ANOVA) will be performed. Repeated measures ANOVA will be used to confirm if there are differences in each group (within-group comparisons), considering each group in isolation, between the five assessments in each of the spinal mobility variables (at baseline, immediately after Abt’s fatigue protocol, immediately after recovery modalities, 15-min after recovery modalities, 30-min after recovery modalities). To calculate between-group differences from baseline assessment to final follow up, a five-way repeated-measures ANOVA will be performed. With the spinal mobility and/or postural stability and/or EMG variables outcome as dependent factor, with five levels corresponding to every time of assessment (t1, t2, t3, t4, and t5), and the two groups (experimental group A vs. experimental group B) as independent factors. The Bonferroni correction will be applied to control for the increased probability of significant findings due to multiple testing. Between- and within-group effect sizes for all quantitative variables will be measured with the Cohen d coefficient: small effect (less than d = 0.2 and ηp2 = 0.01); moderate effect (approximately d = 0.5 and ηp2 = 0.06); and large effect (greater than d = 0.8 and ηp2 = 0.14) (Cohen, 2013). The α-level will be set to 0.05 for all statistical tests.

## 3 Discussion

The present study will address the modeling of trunk muscle fatigue and recovery-related changes in spinal mobility and postural stability in sedentary middle-aged adults at risk of developing non-specific low back pain. People who spend most of their working hours seated, due to their work nature, are more susceptible to developing musculoskeletal disorders (Hanna et al., 2019). Low back pain is the most prevalent occupational musculoskeletal disorder (Csuhai et al., 2020; Zemková et al., 2021c). During prolonged sitting, the static loading of the lumbar spine can cause deep trunk muscles fatigue that may arise from continuous contraction of the muscles (Areeudomwong et al., 2012). Consequently, the neural control subsystem attempts to maintain spinal stability by increasing superficial trunk muscles activation in order to compensate for deep trunk muscles dysfunction (Saiklang et al., 2022). Under fatigue, there is an increased activity of superficial trunk muscles which can reduce the muscular support to the spine and increase stress on ligaments and intervertebral discs. Consequently, it reduces intervertebral disc height, leading to impaired spinal mobility. The reduction of disc height increases the amount of compression on sensitive spinal structures. This may stimulate nociceptor activity, which could be one of the reason for developing pain (Saiklang et al., 2022).

In addition to decrease trunk muscle activity under fatigue, proprioceptive sensitivity also decreases by muscle fatigue (Tajali et al., 2022). The combination of these two factors, can lead to impairment of postural stability (Larson and Brown, 2018). Adverse effect of trunk muscle fatigue on ability of maintain balance can be explained by alternation of neurophysiological mechanisms. Repeated muscular contractions caused by mechanical constraints reduce the activity of muscle spindles (Macefield et al., 1991). Motoneurons activation also reduces under fatigue, which results in decreasing of the discharge frequency of sensorial fibers in muscle spindles (Madigan et al., 2006). Thus, the integration of sensory inputs and the firing patterns of motoneurons are influenced, which leads to fewer motor units being recruited to control postural sway (Gribble and Hertel, 2004). Additionally, the central nervous system does not get accurate information about body location in space at any moment due to variations in proprioceptive input brought on by weariness (Martin et al., 2006). Under fatigue, inputs from group III and IV muscle afferents from antagonist or homonymous muscles decrease extensor motor neurons, whilst flexor motor neurons innervations are facilitated (Martin et al., 2006). Especially the fatigue of lumbar extensor muscles decreases the joint movement sense (Taimela et al., 1999), which initially increases larger lumbar motions and subsequently also postural sways (Pline et al., 2005).

The break is recommended to be applied during prolonged sitting at workplace (Waongenngarm et al., 2018). In particular, active breaks in the form of exercise have a positive effect on the recovery of musculoskeletal discomforts (Maciel et al., 2018), mainly low back pain symptoms in employees (Gobbo et al., 2019). The most frequently used are muscle strengthening and stretching exercises (Shariat et al., 2018; del Pozo-Cruz et al., 2013; Macedo et al., 2011; Sufreshtri and Puspitasari, 2020). However, there are some disadvantages when these exercises are performed at workplace. This includes insufficient time to exercise, financial situation and costs, difficult access to on-site gyms and/or exercise classes, fear of being injured, individualization of exercises, and boredom (Mayer et al., 2013; Skrebutėnaitė and Karanauskienė, 2019). Diaphragmatic breathing exercises seem to be a suitable alternative because they are feasible, effective, safe, affordable, and easy to implement. Due to their simplicity and attractivity, people are more motivated and interested to participate in the exercise program.

Breathing exercises are also beneficial for back health. These exercises have been shown to decrease spinal loading by raising intra-abdominal pressure (Shah et al., 2020). Increased intra-abdominal pressure reduces spine compression force (Kwon et al., 2021). This could play an important role in spinal mobility (Yang et al., 2005). Raising intra-abdominal pressure can also activate the pelvic floor and abdominal wall muscles that improve inferior stability and posterior and anterolateral stability (Rasheed et al., 2021). It helps to stabilize the lumbar spine during static (e.g., standing on tiptoes) and dynamic (e.g., walking with head turns) movements that challenge balance (Ferraro et al., 2020). Moreover, the breathing exercises may increase the strength of the diaphragm and deep core musculature. Muscle firing, and proprioception increase through the diaphragm breathing exercise (Stephens et al., 2017). Both an increase core muscle strength and an improvement in proprioceptive function may contribute to better postural stability. An improvement in diaphragmatic breathing pattern may also be considered as another factor associated with improved balance (Stephens et al., 2017). Despite of the fact that postural stability and spinal mobility are related to low back pain (Mellin, 1987; Mellin, 1990; Karimi and Saeidi, 2013; Thakkar and Kumar, 2015), there are no controlled studies that investigated the acute effect of breathing exercises on these abilities in sedentary middle-aged adults at risk of low back pain.

The management of musculoskeletal problems requires a multidisciplinary approach (Sabo et al., 2016). Starting at the earliest moment possible can increase its effectiveness. Prevention is the first step in the management of low back pain, and physical exercise has a primary effect on it (Schaafsma et al., 2015). However, despite all the efforts in studying the effectiveness of exercise based-break at the workplace on the prevention of musculoskeletal disorders, the focus on people at risk of low back pain is limited. In practice, most people who participate in exercise programs already suffer from some kind of back problem (Džubera et al., 2016). Therefore, focusing on prevention programs can avoid serious chronic musculoskeletal disorders in the future. In addition, more active employees are more productive, require less sick leave, and have overall lower healthcare costs (Taulaniemi et al., 2019). For example, Del Pozo-Cruz et al. (del Pozo-Cruz et al., 2012) found that a web-based exercise program could reduce the social cost of low back pain by €500.00 per episode.

Based on the narrative review by Hamasaki (2020), diaphragmatic breathing can potentially improve respiratory function, reduce stress, anxiety, and depression, and enhance cognitive performance (Hamasaki, 2020). It seems that if diaphragmatic breathing exercises are implemented as an active recovery in the workplace, they could ultimately lead to improved productivity and employee wellbeing. However, performing these exercises as a prevention program for low back pain in employees is rare. The results of our ongoing study will contribute to an understanding of the effects of this technique and its potential application in the prevention of low back pain in employees at the workplace.

We believe that the addition of a diaphragmatic breathing exercises as an active recovery can be successfully implemented in workplace conditions. The evidence allows us to propose that these exercises can help to restore postural stability and spinal mobility in sedentary adults following fatigue of the core muscles induced by prolonged sitting. Proposed recovery modalities could contribute to the improvement of back health by reducing trunk muscles fatigue. Due to the fact that these exercises require very few supplies and can be done almost anywhere, we believe that a large portion of the population could benefit from them.

## Data Availability

The original contributions presented in the study are included in the article/supplementary materials, further inquiries can be directed to the corresponding author.
